# Pathobiology of *Aspergillus Fumigatus* Endophthalmitis in Immunocompetent and Immunocompromised Mice

**DOI:** 10.3390/microorganisms7090297

**Published:** 2019-08-28

**Authors:** Neha Gupta, Pawan Kumar Singh, Sanjay G. Revankar, Pranatharthi H. Chandrasekar, Ashok Kumar

**Affiliations:** 1 Department of Ophthalmology, Visual and Anatomical Sciences, Wayne State University, Detroit, MI 48201, USA; 2 Division of Infectious Disease, Department of Internal Medicine, Wayne State University School of Medicine, Detroit, MI 48201, USA; 3 Department of Biochemistry, Microbiology, and Immunology, Wayne State University, Detroit, MI 48201, USA

**Keywords:** *Aspergillus*, endophthalmitis, eye, retina, inflammation, cytokines

## Abstract

Despite *Aspergillus* being the leading cause of exogenous fungal endophthalmitis following traumatic injury to the eye, its pathogenesis is not fully understood. In the current study, we developed a murine model of *Aspergillus fumigatus* (AF) endophthalmitis and investigated the disease pathobiology. Endophthalmitis was induced by intravitreal injection of *Aspergillus* spores in immunocompetent and immunocompromised (neutropenic) C57BL/6 mice, and disease severity was assessed by eye exam, fungal burden estimation, and histological examination. Our data showed that AF infection caused a time-dependent increase in corneal haze, opacity, and hypopyon beginning at two days post-infection (DPI). The fungal burden in infected eyes of immunocompetent mice peaked at 2 DPI and declined over 9 DPI. AF-infected neuroretina exhibited induction of innate immune response via upregulation of Toll-like receptors (TLRs) and inflammatory mediators (TNFα, IL-1β, and IL6), and increased polymorphonuclear neutrophil (PMN) infiltration. Histological analysis revealed heavy cellular infiltrates in the vitreous cavity as well as disruption of normal retinal architecture and increased retinal cell death. Neutropenic mice exhibited severe disease pathology with the prolonged fungal burden and increased inflammatory mediators. Our study described the first immunocompetent murine model of exogenous AF endophthalmitis and demonstrated an important role of neutrophils in innate defense against fungal endophthalmitis.

## 1. Introduction

Endophthalmitis is a detrimental ocular infection leaving one-third of those infected nearly blind [1]. Severe vision loss is a result of retinal damage caused by both pathogen virulence factors and an uncontrolled host inflammatory response [2,3,4]. After bacterial pathogens [5], fungi are the leading cause of both exogenous and endogenous endophthalmitis [6]. The exogenous fungal endophthalmitis is more common following penetrating ocular trauma, whereas endogenous fungal endophthalmitis results from hematogenous spread of organisms to the eye mainly in immunocompromised individuals [7,8,9]. Fungal endophthalmitis accounts for 8.6 to 18.6% of culture-positive endophthalmitis where *Candida* and *Aspergillus* spp. are the most common causative agents [10,11]. The prognosis of fungal endophthalmitis is poor, depending on the virulence of the pathogen, timing, and mode of intervention.

*Aspergillus* species are ubiquitous saprophytic molds, often affecting immunocompromised patients. However, infection in an immunocompetent individual has been also reported [12]. *Aspergillus* has been reported to cause both exogenous [13] and endogenous [14,15,16] endophthalmitis. In the eye, *Aspergillus* grows preferentially along retinal pigment epithelium and subretinal space resulting in a poor visual prognosis [17]. *Aspergillus* endophthalmitis results in the rapid onset of pain, along with confluent yellowish infiltrate in the macula, choroid and subretinal space, hypopyon in subretinal and subhyaloidal space, retinal hemorrhage, and visual loss [18]. 

Since most data on *Aspergillus* endophthalmitis are limited to clinical and epidemiological studies [19,20], the molecular pathogenesis of this disease is not well understood. This, in part, could be due to limited or unavailability of appropriate fungal endophthalmitis animal models. However, few earlier studies have utilized guinea pig [21] and rabbit [22,23] models to test the therapeutic efficacy of antifungal agents in *Aspergillus fumigatus* (AF) endophthalmitis. Similarly, we evaluated isavuconazole for the treatment of AF endophthalmitis in a mouse model [11]. Thus the development of animal models for *Aspergillus* endophthalmitis can help to address the pathophysiologic process of this disease and the determination of therapeutic efficacy of antifungal drugs. 

In the current study, we describe a murine model of exogenous *A. fumigatus* endophthalmitis and characterize the disease pathogenesis and retinal innate immune response. Better understanding of the protective immune mechanisms evoked in *A. fumigatus* endophthalmitis is likely to lead to newer immunomodulatory approaches to mitigate vision loss. 

## 2. Materials and Methods

### 2.1. Mice and Ethics Statement 

C57BL/6 mice (both male and female mice, 8–12 weeks of age) were purchased from the Jackson Laboratory (Bar Harbor, ME, USA) and housed at the Kresge Eye Institute (Detroit, MI, USA). Animals were maintained in a 12:12 light/dark cycle, and fed LabDiet rodent chow (Labdiet; Pico Laboratory, St. Louis, MO, USA) and water ad libitum. Mice were treated in compliance with the Association for Research in Vision and Ophthalmology (ARVO) Statement for the Use of Animals in Ophthalmic and Vision Research, and all procedures were approved by the Institutional Animal Care and Use Committee (IACUC) of Wayne State University under protocol 16-06-104. 

### 2.2. Preparation of Fungal Spores 

A clinical isolate of *A. fumigatus* was obtained from the Division of Infectious Diseases [11], Department of Internal Medicine, at Wayne State University School of Medicine. For sporulation, *A. fumigatus* was grown on Sabouraud Dextrose Agar (SDA) plates for six days at room temperature. Following sporulation, the spores were harvested, and the count was adjusted to the desired colony forming units (CFU) by diluting the spores in sterile phosphate-buffered saline (PBS).

### 2.3. Induction of Aspergillus Endophthalmitis 

Endophthalmitis was induced in C57BL/6 mice by intravitreal injection of *A. fumigatus* spores (15,000 CFU/µL/eye). The PBS injected eye served as control. At the desired time points post-infection (up to 9 days post-infection (DPI)) clinical examinations were performed using a slit lamp. The ocular disease was graded on a four-point scale, as described previously [24,25]. The enucleated eyes of the infected mouse were subjected to fungal burden estimation, inflammatory cytokine/chemokine assays, polymorphonuclear neutrophil (PMN) infiltration, and histological analysis as described in the following sections.

### 2.4. Fungal Burden Estimation

Fungal burden was estimated using serial dilution and plate count method as described previously [11,26]. Briefly, at each respective time point, the eyes were enucleated and homogenized in sterile PBS by using a Dounce homogenizer. The homogenate was serially diluted in sterile PBS and plated on SDA plates and incubated at 37 °C. Following growth, the fungal colonies were counted and the results were expressed as mean number of CFU/eye ± Standard deviation (SD).

### 2.5. Cytokine/Chemokine ELISA

At desired time points post-infection, eyes were enucleated, and lysates were prepared using TissueLyser II (Qiagen, Valencia, CA, USA). The total protein was estimated using a bicinchoninic acid (BCA) protein assay kit (Thermo Fisher Scientific, Rockford, IL, USA), and 15–20 μg total protein was used for cytokine measurements. The ELISA was performed using commercially available ELISA kits for TNF-α, IL-1β, and IL-6 (BD Biosciences, San Jose, CA, USA) per the manufacturer’s instructions. The data are presented as the mean pg/mg of the tissue lysates ± standard deviation (SD). 

### 2.6. RNA Extraction and qRT-PCR 

Total RNA was extracted from *Aspergillus* infected and control mice retina using Trizol as per the manufacturer’s recommendation (Thermo Scientific, Rockford, IL, USA). cDNA was synthesized using 1 µg of total RNA by Maxima first-strand cDNA synthesis kit, as per the manufacturer’s instructions (Thermo Scientific, Rockford, IL, USA). The cDNA was amplified using gene-specific PCR primers using StepOnePlus™ Real-Time PCR system (Applied Biosystems, Grand Island, NY, USA). All primers and TaqMan probes (Prime Time Mini qPCR Assay) were purchased from Integrated DNA Technologies (Coralville, IA, USA). The quantification of gene expression was determined via the comparative ΔΔCT method. Gene expression in the test samples was normalized to the endogenous control, GAPDH, and was reported as fold change relative to GAPDH gene expression.

### 2.7. Immunofluorescence and TUNEL Staining

Immunostaining was performed on retinal cryosections following *Aspergillus* infection as described in our recent publications [27,28]. Briefly, infected and control mice eyes were enucleated and fixed in 4% PFA, dehydrated, and embedded in paraffin. Thin (10–12 µm) sections were made using a cryostat and mounted onto lysine coated microscope slides. For immunostaining, retinal sections were fixed in 4% PFA for 20 min followed by four washes with PBS (10 min each wash). Retinal sections were permeabilized and blocked with 10% normal goat serum with 0.5% Triton X100 for 2 h at room temperature (RT) and incubated overnight with primary antibody (1:100). The next day, sections were washed four times with PBS (10 min each) and incubated with anti-mouse/rabbit Alexa Fluor 485/594-conjugated secondary antibody (1:200) for 2 h at RT. The retinal sections were washed with PBS (four washes, 10 min each), and the slides were mounted in Vectashield anti-fade mounting medium (Vector Laboratories, Burlingame, CA, USA) and visualized using Eclipse 90i fluorescence microscope (Nikon, Melville, NY, USA).

Terminal deoxynucleotidyl transferase dUTP nick end labeling (TUNEL) was performed on retinal cryosections using an ApopTag^®^ Fluorescein In situ Apoptosis Detection Kit (Millipore, Billerica, MA, USA) as per the manufacturer’s instructions. The TUNEL-stained cells were imaged using an Eclipse 90i fluorescence microscope (Nikon).

### 2.8. Histological Assay

Eyes from the *Aspergillus* infected, and control mice were enucleated at desired time points post-infection for histopathological examination and fixed in 4% formalin. The embedding, sectioning, and hematoxylin and eosin (H&E) staining were performed by Excalibur Pathology, Inc. (Oklahoma City, OK, USA). Histological sections were scanned using a PathScan Enabler IV scanner (Mayer Instruments, Houston, TX, USA).

### 2.9. PMN Infiltration 

Flow cytometry was used to determine the infiltration of PMNs in *Aspergillus* infected retina, as described previously [26,29]. Briefly, at each desired time point post-infection, the retinas were isolated from the eyes and digested with Accumax (Millipore, Billerica, MA, USA) for 10 min at 37 °C. Following digestion, the retinal tissue was triturated by passing through a 23-gauge needle and syringe and filtered through a 40 µm cell strainer (BD Falcon, San Jose, CA, USA). The cells were blocked using Fc Block (BD Biosciences, San Jose, CA, USA) for 30 min, followed by washing with PBS containing 0.5% bovine serum albumin (BSA). Cells were then incubated with CD45-PE-Cy5 and Ly6G-FITC antibodies (BD Biosciences, San Jose, CA, USA) for 30 min in the dark. After subsequent washing steps, the cells were acquired by a BD AccuriC6 flow cytometer (BD Biosciences, Ann Arbor, MI, USA). At least 50,000 cells were analyzed in each treatment. The data were analyzed using AccuriC6 software (BD Biosciences, Ann Arbor, MI, USA).

### 2.10. Neutrophil Depletion 

To make mice neutropenic, Anti-Ly6G-1A8 antibody (200 µg/100 µL/mice) (R&D Systems, Minneapolis, MN, USA) were administered systemically by intraperitoneal (i.p.) injection 24 h before *Aspergillus* infection [30]. Control mice received PBS by i.p. injections.

### 2.11. Statistical Analysis 

All data are expressed as the mean ± standard deviation (SD) unless indicated otherwise. Statistical differences between experimental groups were determined using unpaired Student’s *t*-test and one-way ANOVA. All statistical analyses were performed using GraphPad Prism 8 (GraphPad Software, La Jolla, CA, USA). A value of *p* < 0.05 was considered statistically significant. All experiments were performed at least three times unless indicated otherwise.

## 3. Results

### 3.1. Intravitreal Inoculation of AF Spores Causes Endophthalmitis in C57BL/6 Murine Eyes 

**Figure 1:**
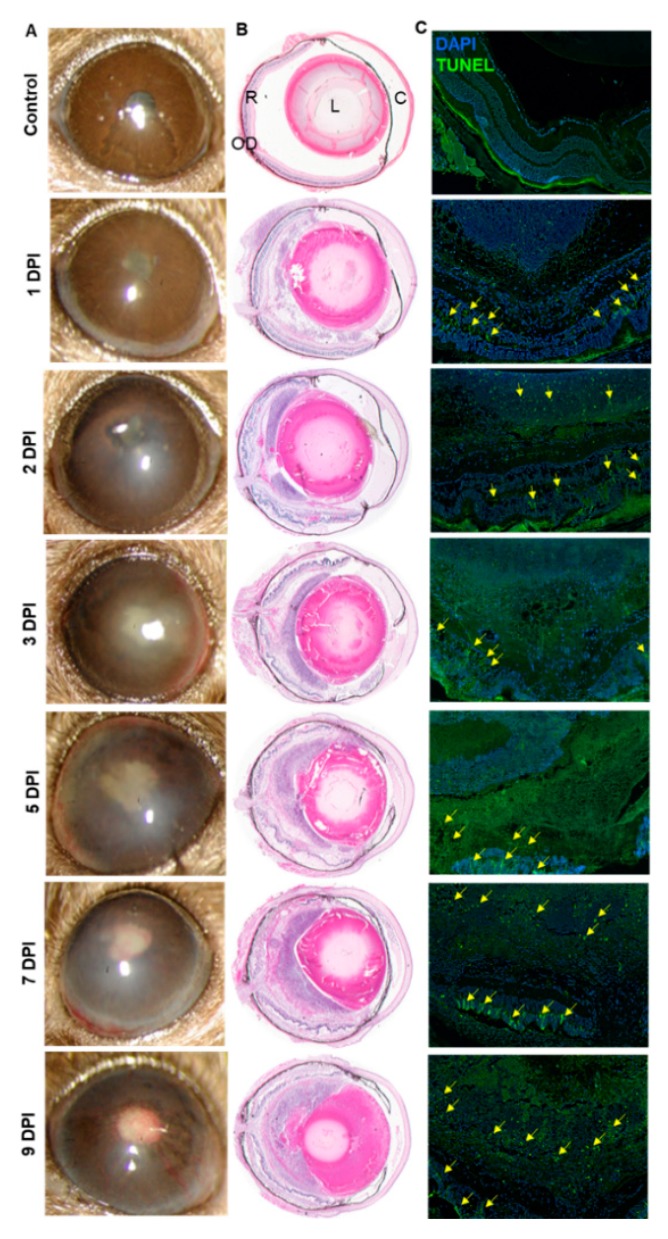
Wild type (WT) C57BL/6 (B6) mice eyes (*n* = 12 each time point) were infected with *Aspergillus fumigatus* spores (15,000 colony forming units (CFU)/eye) by intravitreal injections. Phosphate-buffered saline (PBS) injected eyes were used as control. (**A**) Slit-lamp examination was performed at indicated time points and photomicrographs were taken from representative eyes. (**B**) For histological analysis, eyes were enucleated at indicated time points and subjected to hematoxylin and eosin (H&E) staining. (**C**) To evaluate retinal cell death, retinal cryosections were subjected to TUNEL staining (TUNEL positive cells shown by the yellow arrow) at indicated time points. R: retina, OD: optic disc, L: lens, C: cornea.

In healthy individuals, AF mainly causes exogenous endophthalmitis. To mimic this situation, we performed a dose-response study by injecting AF spores in the eyes of immunocompetent, C57BL/6 (B6) mice. We found that intravitreal injections of 15,000 spores/eye resulted in reproducible fungal endophthalmitis in B6 mice as evidenced by a time-dependent increase in corneal opacity, hypopyon, angiogenesis, and intraocular inflammation (Figure 1A). In contrast, control animals exhibited comparatively clear cornea and anterior chamber with no visible signs of inflammation. To further assess the disease severity, histological analysis was performed which demonstrated a time-dependent increase in cellular infiltration, retinal folding as well as disorganization of the retinal architecture; 5 DPI onwards the AF-infected retina was totally disintegrated (Figure 1B). These findings coincided with increased retinal cell death, as indicated by a greater number of TUNEL positive cells in the infected eyes (Figure 1C). Together, these results indicate that AF causes endophthalmitis and results in severe retinal tissue damage in mouse eyes.

### 3.2. AF Infected Eyes Exhibited a Temporal Decrease in Fungal Burden and Neutrophil Infiltration

**Figure 2:**
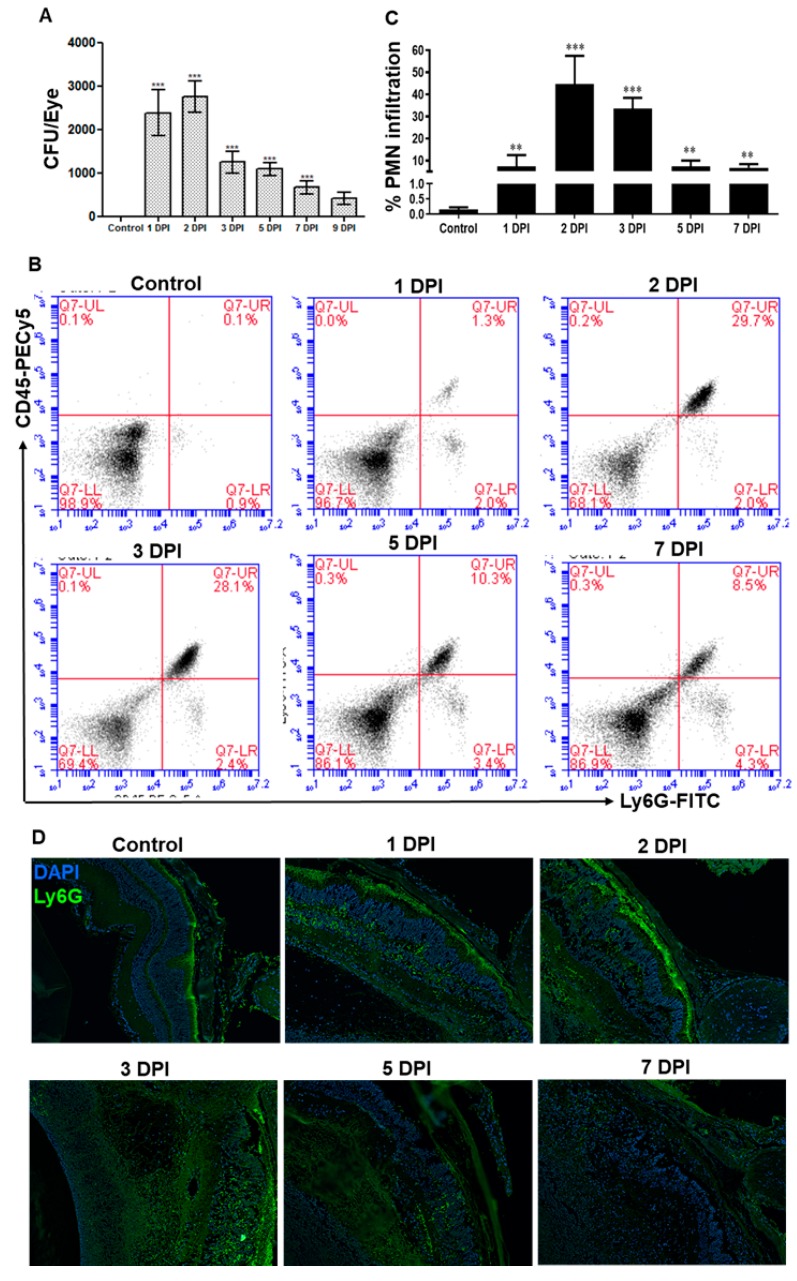
WT B6 mice eyes (*n* = 10 each time point) were infected with *A. fumigatus* spores (15,000 CFU/eye) by intravitreal injections. (**A**) At indicated days post-infection (DPI), eyes were enucleated, homogenized, and the bacterial burden was estimated via serial dilution plating. (**B**) Two retinas were pooled to make single-cell suspensions and the cells were stained with anti-CD45-PE-Cy5 and anti-Ly6G-FITC antibodies for polymorphonuclear neutrophil (PMN) staining. Dot plot showing CD45-Ly6G positive (upper-right quadrant) PMNs. (**C**) Bar graph representing the percent PMN infiltration in the retina using flow-cytometry. (**D**) Retinal cryosections were subjected to anti-Ly6G staining for PMNs. **, *p* < 0.005; ***, *p* < 0.0005; One-way ANOVA.

To determine the fungal growth, serial dilution and plate counting were performed on enucleated eyes at various days post-infection (DPI), and our data showed that fungal burden modestly increased at 2 DPI followed by a gradual decline (Figure 2A). Since neutrophils are the main innate immune cells recruited in response to microbial infection, the flowcytometry analysis was performed to assess neutrophil infiltration (Figure 2B). Similar to the fungal burden, our data showed that neutrophil infiltration peaked at 2 DPI and declined at later time points (3–7 DPI), but remains significantly higher as compared to uninfected control eyes (Figure 2C). Immunostaining also confirmed the presence of PMNs in infected mouse retinal tissue, as evidenced by increased staining of Ly6G positive cells (Figure 2D). These results indicate a direct correlation between fungal load and increased neutrophil infiltration in AF endophthalmitis.

### 3.3. AF Infected Retina Exhibited Increased Inflammatory Mediators and Induced Expression of Toll-Like Receptors (TLRs)

**Figure 3:**
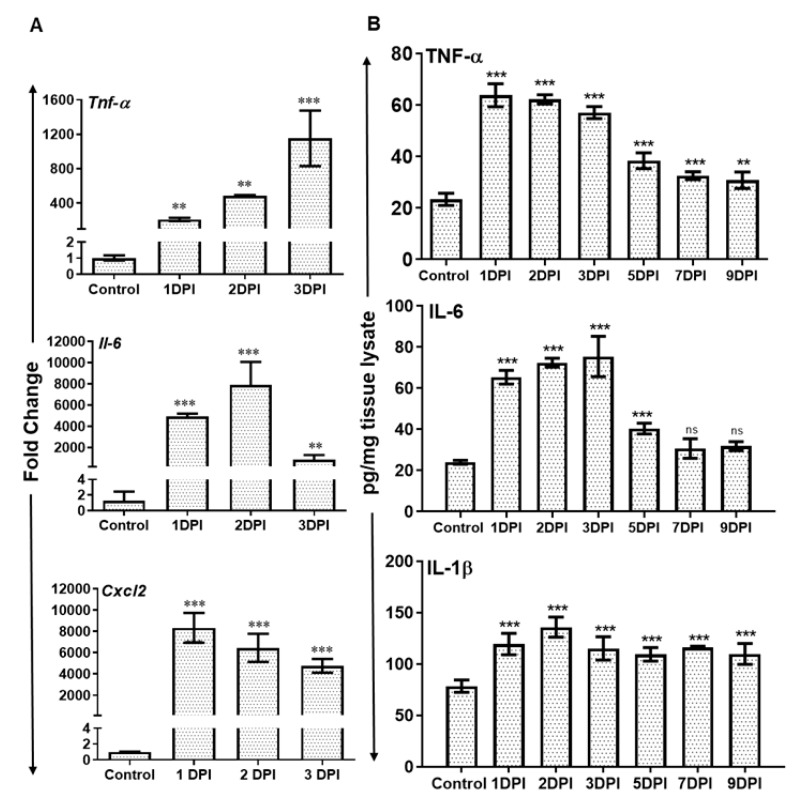
WT B6 mice eyes (*n* = 10 each time point) were infected with *A. fumigatus* spores (15,000 CFU/eye) by intravitreal injections. (**A**) The neural retina was used for RNA isolation and qRT-PCR for inflammatory cytokines/chemokines *Tnfα*, *Il-6*, and *Cxcl2*. (**B**) The eye lysates from infected and control eyes were subjected to ELISA to quantify the protein level of indicated inflammatory cytokines. ns, not significant, **, *p* < 0.005; ***, *p* < 0.0005; One-way ANOVA.

The recruitment of neutrophils is a dynamic process regulated by the production of inflammatory chemokine and cytokines. Therefore, we assessed the expression of various inflammatory mediators at transcripts as well as protein levels. The qRT-PCR analysis revealed significantly increased mRNA levels of several inflammatory cytokines and chemokines e.g., *Tnf α, Il-6*, and *Cxcl2* in AF-infected retina (Figure 3A). *Tnf α* transcripts showed time-dependent increased expression while the transcripts of *Il-6* and *Cxcl2* peaked at 2 and 1 DPI respectively and slightly declined thereafter.

We confirmed the translation of these mRNA transcripts by measuring the protein levels of inflammatory cytokines by ELISA. Our results show that *Aspergillus* infection resulted in the induction of inflammatory mediators TNFα, IL-1β, and IL-6 at protein level as well (Figure 3B). The expression of these cytokines peaked at 1 or 2 DPI and remained elevated until 3 DPI, followed by a slight decline thereafter.

**Figure 4:**
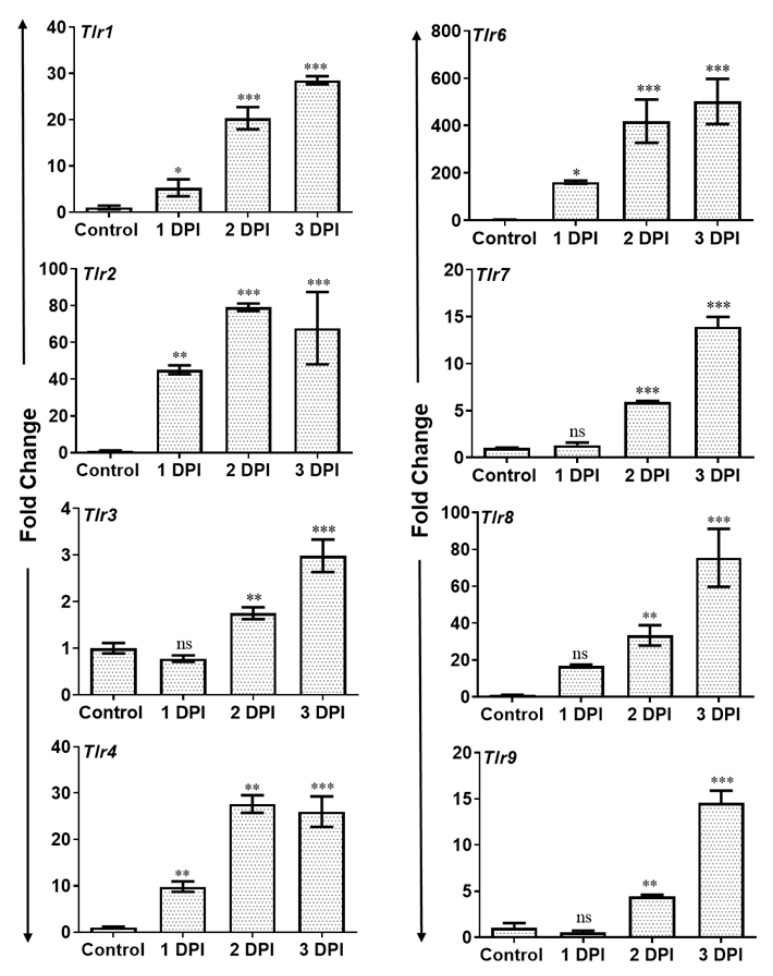
WT B6 mice eyes (*n* = 10 each time point) were infected with *A. fumigatus* spores (15,000 CFU/eye) by intravitreal injections and the vitreous/retina were isolated at the indicated time points. Infected and control neural retina were used for RNA isolation and subjected to qRT-PCR for various Toll-like receptors (*Tlr*1–9) at indicated DPI. The data are presented as fold changes in comparison with uninfected controls. ns = not significant, *, *p* < 0.05; **, *p* < 0.005, ***, *p* < 0.0005; One-way ANOVA.

Activation of Toll-like receptors (TLRs) following pathogen challenge plays an important role in the initiation of the early innate immune response against microbial infections. Since we observed PMN infiltration and induction of inflammatory mediators in AF-infected eyes, we assessed the expression of TLRs using qRT-PCR. To this end, our results show that AF infection resulted in a significant and time-dependent induced expression of mRNA transcripts of several TLRs including TLR1, 2, 3, 4, 6, 7, 8, and 9 in infected mouse retina (Figure 4). 

### 3.4. Neutropenic Mice Are More Susceptible to AF Endophthalmitis 

**Figure 5:**
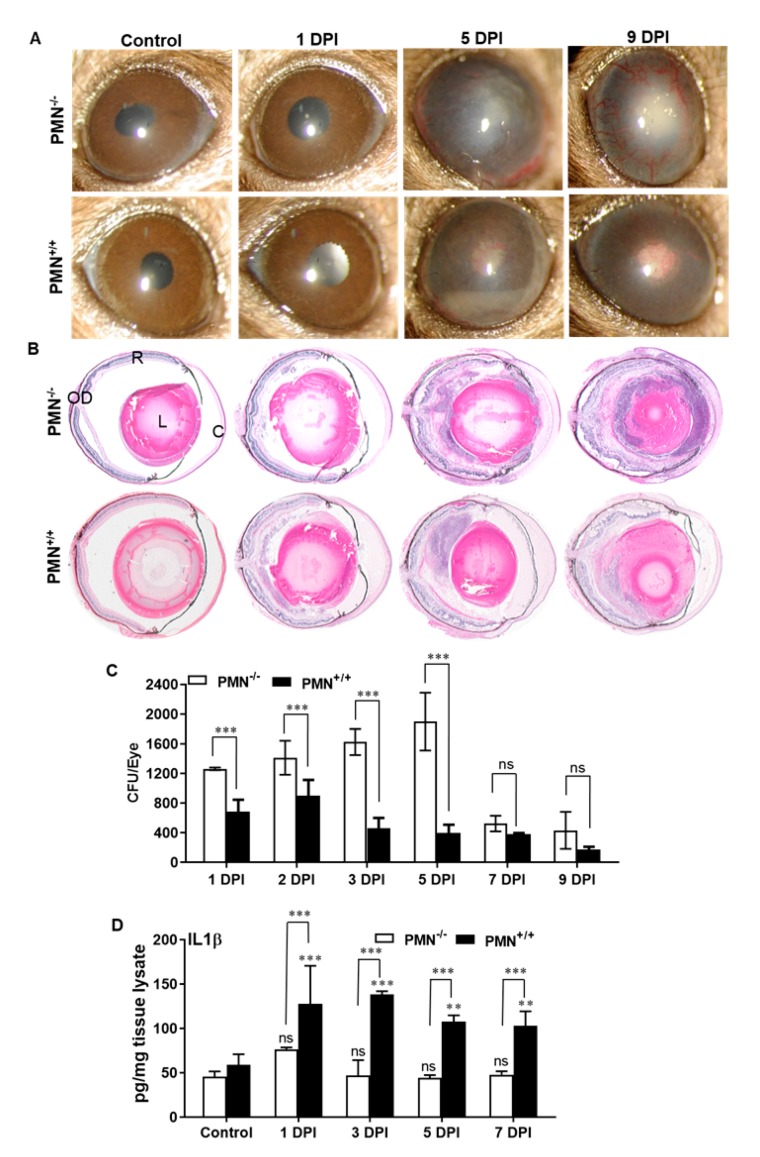
WT B6 mice were made neutropenic (PMN^−/−^) by intraperitoneal injection of Anti-Ly6G-1A8 antibody. Immunocompetent B6 mice (without PMN depletion, PMN^+/+^) were used as control. Then, 24 h following neutropenia eyes (*n* = 10 each time point) were infected with *A. fumigatus* spores (15,000 CFU/eye) by intravitreal injections. (**A**) Slit-lamp examination was performed at indicated time points and photomicrographs were taken from representative eyes. (**B**) For histological analysis, eyes were enucleated at indicated DPI and subjected to H&E staining. (**C**) Fungal burden was estimated from eye lysates via serial dilution plating. (**D**) The eye lysate was subjected to ELISA for representative inflammatory cytokine e.g., IL-1β. ns, not significant; **, *p* < 0.005; ***, *p* < 0.0005; One-way ANOVA. R: retina, OD: optic disc, L: lens, C: cornea.

Since we observed increased PMN infiltration correlating with the fungal burden in AF-infected eyes, we sought to determine the specific role of PMNs in this model. As we reported earlier, mice were made neutropenic using systemic injections of an anti-Ly6G-1A8 antibody to specifically deplete PMNs [30]. Our results show that neutropenic C57BL/6 mice sustained more severe AF endophthalmitis as revealed by increased hypopyon, anterior chamber haze, corneal opacity, as well as angiogenesis in infected eyes (Figure 5A). Histological analysis also corroborated with increased retinal tissue damage in neutropenia versus immunocompetent mice (Figure 5B). Fungal burden estimation revealed that immunocompetent mice had a slight increased fungal burden at 2 DPI followed by decline up to 9 DPI. In contrast, the fungal burden remained elevated up to 5 DPI in neutropenic mice, but no significant difference was observed at 7 and 9 DPI time points (Figure 5C). Interestingly, the levels of inflammatory mediators (e.g., IL-1β) remained higher at all time points in neutropenic versus immunocompetent mice (Figure 5D). These results indicate an essential role of PMNs in protection against fungal endophthalmitis.

## 4. Discussion

Over the past decades, the incidence of ocular fungal infections has increased due to extended use of cancer chemotherapy, immunosuppressive agents, long-term broad-spectrum antibiotics, and increasing number of immunocompromised patients with increased incidence of diabetes, cancer, and HIV infection [31,32]. Among ocular mycoses, corneal infection (keratitis) remains the most frequent presentation. Therefore, extensive studies, including the pathobiology are available for fungal keratitis. However, fungi have the ability to infect multiple ocular structures such as conjunctiva, eyelid, lacrimal gland, orbit, eyelid, sclera, and intraocular structures such as uvea and the retina. The delicate nature of intraocular tissues such as the retina, lends itself to significant visual disability, blindness, and even enucleation when challenged by fungal infections, like endophthalmitis. Considering the importance of understanding the molecular pathogenesis of fungal endophthalmitis, in this study, we demonstrated the pathological changes in *Aspergillus* infected murine eyes and the induction of retinal innate responses. Moreover, using neutropenic mice, our study elucidated the protective role of neutrophils in fungal endophthalmitis. 

*Aspergillus* has the ability to cause both exogenous and endogenous endophthalmitis, in immunocompetent and immunocompromised individuals respectively [14,33,34,35,36,37,38]. Here, we mimicked exogenous fungal endophthalmitis by directly inoculating *Aspergillus fumigatus* (AF) spores in the eyes of immunocompetent C57BL/6 wild type mice. We observed that AF-infected eyes show the characteristics of endophthalmitis including the development of corneal haze, opacity, hypopyon, and corneal neovascularization. Furthermore, heavy cellular infiltrates, fibrin deposits and retinal tissue damage were evident in infected eyes. The retinal tissue integrity began to deteriorate 2 days post-infection (DPI) and by day 5 retinal architecture was completely disintegrated with increased retinal cell death. Similar disease pathology has been reported in severe cases of bacterial endophthalmitis including *Staphylococcus aureus*, *Bacillus cereus*, and *Acinetobacter baumannii* [26,30,39,40,41]. Heavy corneal angiogenesis has also been reported in *Candida albicans* model of keratitis [42]. When we measured the fungal burden in infected eyes, we found the fungal burden peaked at 2 DPI and reduced thereafter. Despite the reduction in the fungal burden, we observed a time-dependent increase in angiogenesis and severe histological damage in the eye indicating that host-mediated inflammatory signals lead to the destruction of retinal tissue and vision in fungal infection. This trend correlated with *A. baumannii* endophthalmitis, where the bacterial growth has been reported to decrease by 3 DPI, but ocular damage increases in a time-dependent manner [43]. 

TLRs have been shown to play a key role in the initiation of innate immune defense in the retina following bacterial and viral infection and TLR deficiency makes mice more susceptible to disease [26,27,28,40,44,45,46,47,48]. Several fungal ligands have been shown to interact with various TLRs, e.g., fungal chitin directly binds to TLR2 and induce an inflammatory response [49]. Similarly, fungal-β-glucans, phospholipo-mannans and linear beat-1,2-oligomannoside are also recognized by TLR2 [50]. TLR4 has been reported to be activated by *Candida albicans* O-linked mannans as well as *Cryptococcus neoformans* glucuronoxylomannan [50]. TLR2 has been shown to be activated by unidentified ligands present on both conidia and hyphae forms of *A. fumigatus* whereas ligands for TLR4 have been reported only on conidial forms [50,51,52]. To investigate the potential involvement in *Aspergillus* induced host innate immune response we measured the expression of various TLRs in the retina. We showed that *Aspergillus* infection induced the innate immune response via activation of several TLRs including TLR1, 2, 3, 4, 6, 7, 8, and 9 in the retina. Our findings corroborated with studies showing initiation of immune response via TLR2 and TLR4 in murine macrophages [53] and Human PBMC [54] in vitro following *Aspergillus* challenge, as well as in experimental invasive pulmonary aspergillosis [55]. Further studies involving the knockdown of specific TLRs are needed to delineate their role. 

TLR activation leads to the production of inflammatory cytokines and chemokines which recruit PMNs and control the growth of the pathogen. We observed that *Aspergillus* infection induced the production of several inflammatory cytokines and chemokines including TNF-α, IL-1β, IL6, and CXCL2 in the eyes. This observation corroborated with the findings in bacterial endophthalmitis and keratitis models [26,29,30,40,44,56]. Inflammatory cytokine TNFα is an early responder compared to other cytokine/chemokines, and its upregulation is in parallel with the influx of PMNs [57]. We observed an increase in CXCL2 chemokine expression which is also known to be regulated by TNFα upregulation and another contributor to the rise in PMNs [58]. Experimental models have identified PMNs as the primary infiltrating cell type during bacterial ocular infections [57,59] as well as *Aspergillus* keratitis [60,61], and our current data support these studies by showing heavy PMN infiltration in the case of *Aspergillus* endophthalmitis as well. We observed that in the *Aspergillus* infected retina, PMN recruitment began at 1 DPI, peaked at 2 DPI, and slightly declined thereafter. Yet, the recruitment and activation of neutrophils within an infected eye is a biological dilemma. PMN infiltration is the immediate response for acute intraocular infection, but the generation of toxic reactive oxygen intermediates and other inflammatory mediators by PMNs may results in bystander damage to delicate tissues of the retina [57,58]. It was also found that the injection of TNFα into the vitreous of rabbits and rats have induced vascular permeability, stimulates mononuclear phagocytes and activates proinflammatory signaling cascades [57,62,63]. Ramadan et al. reported TNFα- knockout mice infected with *B. cereus* faced a more rapid decline in retinal function, increased the bacterial burden, and lowered PMN count [57]. Hence, with the combination of PMN infiltration and inflammatory cytokine release, retina are more prone to damage. In the current study, we found the peak of inflammatory mediators and PMNs at day 2 or 3 following which there is the most retinal damage despite the reduction in fungal burden, as seen with the increased retinal folds, tissue destruction, and heavy angiogenesis. 

Fungal ocular infection has been reported more frequently in immunocompromised individuals and, in general, neutropenic patients are at particularly high risk for *A. fumigatus* infection [12]. Here, we investigated the role of immunocompromisation by PMN depletion in *A. fumigatus* endophthalmitis. Our study reveals that neutropenia (PMN^−/−^) led to increased severity of *Aspergillus* endophthalmitis as revealed by increased corneal haze, neovascularization with angiogenesis, and destruction of retinal architecture as compared to immunocompetent (PMN^+/+^) mice. PMN^−/−^ mice also exhibited increased fungal burden in the eyes as compared to PMN^+/+^ mice, indicating that limited PMN infiltration is required for fungal clearance. We also showed that neutropenic mice exhibited reduced cytokine response which initially may be required for pathogen clearance, and this might have led to increased severity of disease. 

In conclusion, our study demonstrated *Aspergillus* endophthalmitis acquired by exogenous route initiated host innate immune response by activation of several TLRs, followed by induction of inflammatory cytokines and PMN recruitment. Neutropenia makes the mouse more susceptible towards the disease. To the best of our knowledge, this is the first murine model of *Aspergillus*-induced endophthalmitis in both immunocompetent and immunocompromised (neutropenic) mice. Further detailed studies are required to investigate the role of individual TLRs in this disease pathogenesis, and to understand the innate immune response can contribute to better management of patients affected by *Aspergillus* endophthalmitis.
